# Resistance Exercise Training Improves Metabolic and Inflammatory Control in Adipose and Muscle Tissues in Mice Fed a High-Fat Diet

**DOI:** 10.3390/nu14112179

**Published:** 2022-05-24

**Authors:** Pauline S. Effting, Anand Thirupathi, Alexandre P. Müller, Bárbara C. Pereira, Diane M. Sepa-Kishi, Luis F. B. Marqueze, Franciane T. F. Vasconcellos, Renata T. Nesi, Talita C. B. Pereira, Luiza W. Kist, Maurício R. Bogo, Rolando B. Ceddia, Ricardo A. Pinho

**Affiliations:** 1 Faculty of Sports Science, Ningbo University, Ningbo 315211, China; paulinese@gmail.com; 2 Graduate Program in Health Science, Medical School, Universidade do Extremo Sul Catarinense, Criciúma 88806-000, SC, Brazil; barbara_cst@hotmail.com; 3 Graduate de Pós-graduação em Farmacologia, Universidade Federal de Santa Catarina, Florianópolis 88020-302, SC, Brazil; alexandrep.muller@gmail.com; 4 Health Research Centre, School of Kinesiology and Health Science, York University, Toronto, ON M3J 1P3, Canada; dianesepakishi@gmail.com (D.M.S.-K.); roceddia@yorku.ca (R.B.C.); 5 Laboratory of Exercise Biochemistry in Health, Graduate Program in Health Sciences, School of Medicine, Pontifícia Universidade Católica do Paraná, Curitiba 80215-901, PR, Brazil; luis_marqueze@hotmail.com (L.F.B.M.); francianevascocellos@hotmail.com (F.T.F.V.); retiscoski@gmail.com (R.T.N.); 6 Graduate Program in Cellular and Molecular Biology, School of Health and Life Sciences, Pontifical Catholic University of Rio Grande do Sul, Porto Alegre 90619-900, RS, Brazil; talitapereira@gmail.com (T.C.B.P.); lwkist@gmail.com (L.W.K.); mbogo@pucrs.br (M.R.B.); 7 Graduate Program in Medicine and Health Sciences, School of Medicine, Pontifical Catholic University of Rio Grande do Sul, Porto Alegre 90619-900, RS, Brazil

**Keywords:** physical exercise, ladder-climbing training, inflammation, insulin resistance, obesity

## Abstract

This study investigates whether ladder climbing (LC), as a model of resistance exercise, can reverse whole-body and skeletal muscle deleterious metabolic and inflammatory effects of high-fat (HF) diet-induced obesity in mice. To accomplish this, Swiss mice were fed for 17 weeks either standard chow (SC) or an HF diet and then randomly assigned to remain sedentary or to undergo 8 weeks of LC training with progressive increases in resistance weight. Prior to beginning the exercise intervention, HF-fed animals displayed a 47% increase in body weight (BW) and impaired ability to clear blood glucose during an insulin tolerance test (ITT) when compared to SC animals. However, 8 weeks of LC significantly reduced BW, adipocyte size, as well as glycemia under fasting and during the ITT in HF-fed rats. LC also increased the phosphorylation of Akt_Ser473_ and AMPK_Thr172_ and reduced tumor necrosis factor-alpha (TNF-α) and interleukin 1 beta (IL1-β) contents in the quadriceps muscles of HF-fed mice. Additionally, LC reduced the gene expression of inflammatory markers and attenuated HF-diet-induced NADPH oxidase subunit gp91phox in skeletal muscles. LC training was effective in reducing adiposity and the content of inflammatory mediators in skeletal muscle and improved whole-body glycemic control in mice fed an HF diet.

## 1. Introduction

Several experimental studies have shown that aerobic exercise (AE) [1,2,3,4,5,6] and resistance training (RT) [7,8,9] are effective non-pharmacological approaches to treating and preventing obesity and its related metabolic disorders. AE of submaximal intensity (45 to 65% of VO_2_ max) and prolonged duration (30 to 60 min) has the potential to consume a significant amount of glucose and fatty acids for energy production, whereas RT of either low-to-moderate intensity (50% to 75% 1RM) or high intensity (>75% 1RM) is mostly anaerobic and increases the consumption of glucose for energy production [10]. Thus, although imposing distinct metabolic demands on skeletal muscles, both AE and RT can favor weight loss and glycemic control [10,11]. These exercise interventions help maintain metabolic rate and lean mass during weight loss by potentially counteracting energy-sparing mechanisms that are activated under weight-loss conditions [12]. This is significant because any decrease in metabolic rate resulting from reduced adiposity can make it difficult to sustain long-term weight loss [13]. However, even when not associated with weight loss, AE and RT have significantly improved glycemic control in individuals with Type 2 diabetes mellitus (T2DM) [14]. Multiple mechanisms have been reported to regulate glucose utilization during exercise. However, AMP-activated protein kinase (AMPK) [15] and the actin cytoskeleton-regulating GTPase Rac1 [16] have been reported to act as major factors underlying contraction-stimulated glucose uptake in skeletal muscles, thus revealing AMPK and Rac1 as crucial components of the complex molecular machinery by which different exercise modes can effectively manage T2DM.

In addition to their metabolic effects, AE and RT have also been shown to reduce chronic inflammation associated with obesity [17,18,19,20,21,22] and induce changes to the redox profile. Although transient reactive oxygen species (ROS) production by physiological stimuli can be beneficial, chronic ROS generation associated with obesity-induced hyperglycemia/hyperlipidemia can promote insulin resistance and the development of diabetic micro- or macrovascular complications [23,24]. The mechanism by which hyperglycemia-associated ROS production can lead to diabetic angiopathy has been, at least partially, attributed to the diacylglycerol (DAG)-induced protein kinase C (PKC)-dependent activation of NADPH oxidase [23]. In this context, RT decreases cellular oxidant production and contributes to redox rebalancing [18]. Ceramides and oxidative stress, which are elevated in obesity and insulin resistance, also negatively affect insulin signaling and GLUT4 translocation in skeletal muscle cells [25]. However, ceramides and oxidative have been proposed to impair insulin-mediated GLUT4 translocation in skeletal muscle cells by independently affecting Rac–GTP loading and AKT phosphorylation, respectively, [25]. Thus, because skeletal muscles make up approximately 30% and 40% of total body weight in women and men [26], respectively, and play a crucial role in regulating glucose and lipid metabolism [27], alterations in the redox state in this tissue are expected to affect whole-body glycemic control significantly.

Several studies have already demonstrated that exercise is an effective intervention to counteract the deleterious metabolic effects of diet-induced obesity and insulin resistance in rodents, where treadmill running [28], voluntary wheel running [29], and swimming [30] have been the typical modes of exercise utilized in most rodent studies. Much less has been done with resistance exercise in rodents because of the challenges of developing a protocol for rodents that resembles typical resistance training. In this study, we were able to test the effects of resistance training on adiposity, insulin resistance, and oxidative stress by applying ladder climbing (LC) as a mode of resistance exercise.

In this context, we hypothesized that improved insulin sensitivity through RT is accompanied by positive alterations in the redox state that attenuate inflammation in skeletal muscles under conditions of diet-induced obesity. To test this hypothesis, we exposed HF-fed mice to an LC training protocol demonstrated to mimic the effects of RT in humans [31] and also alter the redox state of skeletal muscles [32].

Here, we investigate the effects of LC training on whole-body glycemic control, weight gain, adiposity, and obesity-induced oxidative stress and inflammation in skeletal muscles. Additionally, we explore whether enhanced AMPK and AKT phosphorylation and Rac1 expression are also affected in the skeletal muscles of mice exposed to HF-diet-induced obesity.

## 2. Materials and Methods

### 2.1. Animals, Diet, and Ethics Approval

Male Swiss mice (40 days old, 35.54 g ± 3.14 g) were housed collectively (up to 7 animals/cage) on a 12/12 h light/dark cycle at 22 °C. The animals were divided into two initial large groups: standard chow (SC, *n* = 11; 5 and 6 animals placed in two boxes) and high-fat diet (DIO, *n* = 13; 6 and 7 animals placed in two boxes). Animals were fed ad libitum for the entire duration of the study (26 weeks) either standard chow (SC: 50%, 27%, and 23% of calories provided by carbohydrates (starch and sugars), proteins, and lipids (animal fat and soybean oil), respectively, with the energy density of 3.3 kcal/g) or a high-fat (HF) diet (26%, 15%, and 59% of calories provided by carbohydrates (starch and sucrose), proteins, and lipids (animal fat and soybean oil), respectively, with the energy density of 5.3 kcal/g). The SC was purchased from Pure Animal Nutrition (Cat. PuroLab 22PB; Santo Augusto, RS, Brazil), and the HF diet ingredients were purchased from PragSolutions Bioscience, Jaú, SP, Brazil.

**Figure 1:**
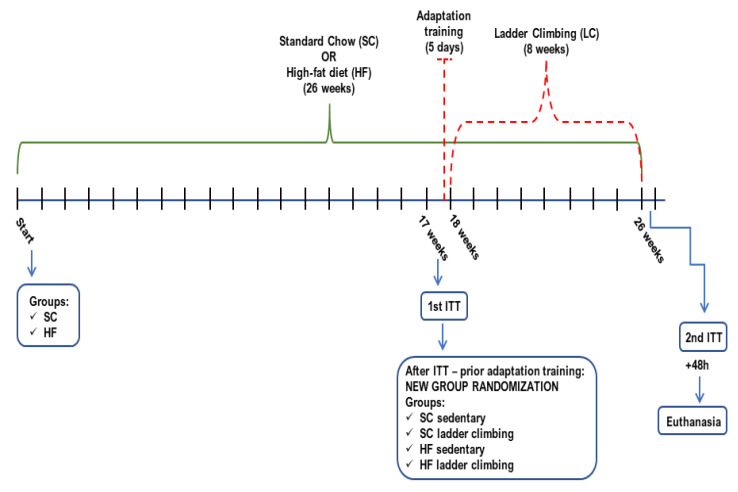
Timeline depicting the experimental design (SC- standard chow; HF- high-fat; ITT- insulin tolerance test).

After 17 weeks, the animals were randomly assigned to resistance physical training (RT) or sedentary groups: SC-fed Sedentary (SC_Sed_, *n* = 5), HF-fed Sedentary (HF_Sed_, *n* = 6), SC plus 8-wk RT (SC_LC_, *n* = 6), and HF plus 8-wk RT (HF_LC_, *n* = 7) (Figure 1). The groups were kept separate in specific boxes to avoid conflict or stress. All procedures were performed according to the Brazilian guidelines for the use of animals in research and approved by the local Ethics Committee (protocol #067/2014-2).

### 2.2. Resistance Training—Ladder Climbing Protocol

The training sessions were performed between 5 pm and 7 pm on a 1 m climbing ladder with a 2 cm distance between the steps and 85° inclination (adapted from Hornberger & Farrar, 2004) [31]. After the 17-wk diet period, the mice first completed a 5-day adaptation protocol that involved one training session per day, with animals completing one climb of the ladder per session (no-load). The session was considered satisfactory when the animal completed the climb from the base to the top of the ladder. The 8-wk LC protocol was adapted from Scheffer et al. (2012) [32] and Vilela et al. (2017) [33] and started three days after the adaptation protocol. Twenty-eight training sessions were performed over eight weeks, with 48 h intervals between sessions. The intensity of LC was progressively increased by attaching weights (20% to 75% of BW) to the animals’ tails. The number of sets was also progressively increased from 5 to 10, with rest intervals of 2 min between sets. The animals climbed five times from the base to the top of the ladder in each group, with no interval between each climb. The LC protocol consisting of loads and sets was as follows: Week 1—20% and 5; Week 2—20% and 7; Week 3—50% and 5; Week 4—50% and 7; Week 5—50% and 10; Week 6—50% and 10; Week 7—75% and 7; Week 8—75% and 10.

### 2.3. Body Weight (BW) and Insulin Tolerance Test (ITT)

BW was measured at the beginning of the study at weeks 2, 4, 8, 12, and 17 and during the RT period at weeks 18, 22, and 26. Two ITTs were performed over the course of the study: (1) at week 17 prior to randomization into the RT intervention and (2) 48 h after the last exercise session at week 26. For the ITT, mice fasted for 6 h, and an initial blood glucose measurement was recorded via tail snip [34]. The animals were then intraperitoneally injected with insulin (2 U/kg), and blood glucose was measured at 5, 10, 15, 20, 25, and 30 min post-injection [35]. Animals with glucose values below 30 mg/dl during the test were kept warm and received intraperitoneal glucose. Blood glucose concentration was measured using a glucometer, and all values were reported in mg/dl. The area under the curve (AUC) was calculated for the post-insulin injection period.

### 2.4. Euthanasia and Sample Processing

Forty-eight hours after the last ITT, animals were euthanized by decapitation, and tissues were harvested. Quadriceps and gastrocnemius (red central portion) muscles were extracted, washed in 0.9% saline, flash-frozen in liquid nitrogen, and stored at −80 °C for subsequent analysis. An inguinal subcutaneous white adipose tissue (WAT) sample was embedded in 4% formalin for subsequent histological analysis.

### 2.5. Western Blot Analysis

Gastrocnemius tissue (~50 mg) was homogenized in lysis buffer (135 mM NaCl, 1 mM MgCl_2_, 2.7 mM KCl, 20 mM Tris base (pH 8), 1% Triton, 10% glycerol, 10.27 mM Na_3_VO_3_, 3.5 mM PMSF, 1 µM aprotinin, 10 mM Na_4_P_2_O_7_). Homogenates were centrifuged, the infranatant was collected, and an aliquot was used to measure protein by the Bradford method [36]. Samples were diluted 1:1 (*vol*:*vol*) with 2x Laemmli sample buffer (Bio-Rad cat.1610737), heated to 95 °C for 5 min, subjected to SDS-PAGE (60 µg of protein), and transferred to PVDF membranes. The membranes were subsequently probed with the following primary antibodies: AKT1/2/3 total (62 kDa—Abcam cat # 126811), phospho-AKT (Ser473—60 kDa—Santa Cruz cat # 7985-R), GSK-3β total (46 kDa—Santa Cruz cat # sc-9166), phospho-GSK-3β (Ser9—46 kDa—Invitrogen cat # MA5-14873), AMPKα total (63 kDa—Santa Cruz cat # 74461), phospho-AMPKα (Thr172) (63 kDa—Santa Cruz cat # 33524). Blots were visualized using chemiluminescence and were scanned directly into an image quantification program (Scion Image^®^). Values were obtained by dividing the values of the phosphorylated protein of interest by its non-phosphorylated content. Values are expressed in arbitrary units (AUs).

### 2.6. RNA Isolation and Quantitative PCR (RT-qPCR)

Molecular analysis of Rac1, AMPKα2, and CaMKK2 β gene expression was performed following the Minimum Information for Publication of Quantitative Real-Time PCR Experiments (MIQE) Guidelines for RT-qPCR experiments [37,38]. Total RNA was isolated from gastrocnemius muscles using TRIzol^®^ Reagent (Thermo Fisher Scientific, Waltham, MA, USA), and cDNA was synthesized from 2 μg of extracted RNA with the High-Capacity cDNA Reverse Transcription Kit (Applied Biosystems™) according to the manufacturer’s instructions. Quantitative PCR was performed using SYBR^®^ Green I (Thermo Fisher Scientific, Waltham, MA, USA) on the 7500 Real-time PCR System (Applied Biosystems, Foster City, CA, USA) using the following amplification conditions: 95 °C (5 min), 40 cycles of 95 °C (15 s), 60 °C (35 s), and 72 °C (15 s). At the end of the cycling protocol, a melt-curve analysis was included (fluorescence measured from 60 to 99 °C) to confirm the specificity of primers and the absence of primer-dimers. All real-time assays were carried out in quadruplicates. *Ppia* was used as a reference gene for normalization [39,40,41]. Relative mRNA expression levels were determined by the 2^−ΔΔCq^ method [37,42], and the results are presented as relative mRNA expression. Sequences of primers can be found in Table 1.

### 2.7. Histological Analysis

The Ing WAT was cross-sectioned and immediately immersed in 4% paraformaldehyde fixative solution and buffered for 48 h before histological processing. The material was embedded in paraffin and cut into a microtome to obtain five-micrometer-thick sections. The slides were stained with hematoxylin and eosin (H&E) staining. A Nikon inverted microscope was used for image acquisition and analysis.

### 2.8. Inflammatory Parameters

TNF-α and IL1β contents were determined in quadriceps muscles using commercial ELISA kits (TNF-α: Invitrogen cat # 887340; IL1β: Invitrogen cat # 88-7013) according to the manufacturer’s instructions. Results were expressed in pg/mg of protein [45].

### 2.9. Statistical Analyses

The Shapiro–Wilk normality test was applied, and all data met the assumptions for normal distribution. Data were expressed as average ± standard deviation (SD), and the significance of differences was calculated using Student’s *t*-test, two-way repeated-measures ANOVA, or two-way ANOVA, followed by Bonferroni posthoc tests, as indicated in the legends of the figures. Statistical significance was set at *p* ≤ 0.05. Graph Pad Prism software version 9 was used for all analyses.

## 3. Results

### 3.1. Effects of Diet on Body Weight (BW), Fasting Blood Glucose, and Insulin Tolerance Parameters

**Figure 2:**
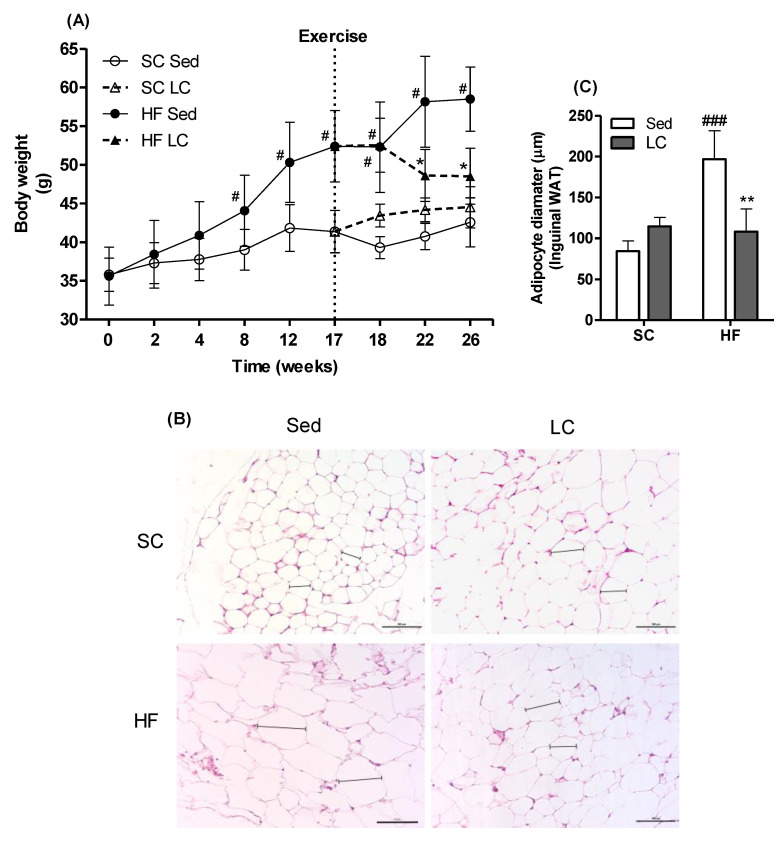
Effects of 17 weeks of HF feeding, followed by 8 weeks of LC on body weight (**A**) and white adipose tissue (WAT) (**B**,**C**). Two-way repeated-measures ANOVA with Bonferroni posthoc test (*n* = 5) (**A**). Representative images of histological photomicrographs of inguinal WAT (*n* = 3) (**B**). Two-way ANOVA with Bonferroni posthoc test. (**C**). * *p* < 0.05, ** *p* < 0.01 vs. respective sedentary (Sed); ^#^ *p* < 0.05, ^###^ *p* < 0.001 vs. respective standard chow (SC) group.

**Figure 3:**
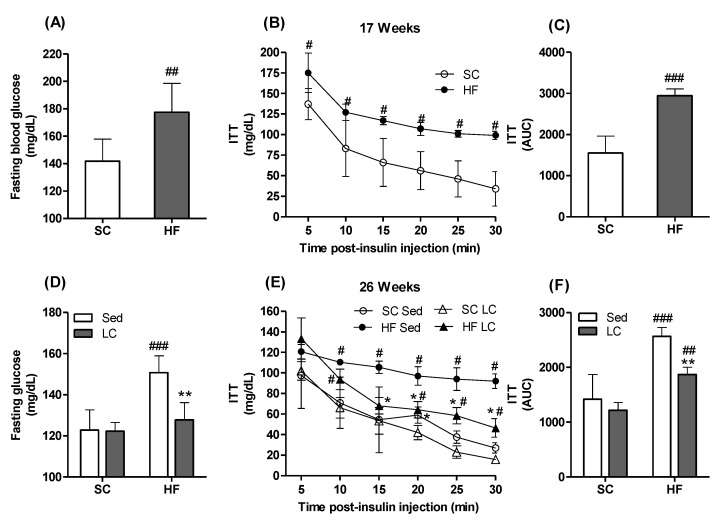
Effects of 17 weeks of HF feeding, followed by 8 weeks of LC on fasting glycemia (**A**) and (**D**), insulin-stimulated blood glucose clearance (insulin tolerance test, ITT, (**B**,**E**) and respective areas under the curve (**C**,**F**). Student´s t-test (**A**–**C**). Two-way ANOVA with Bonferroni posthoc test (**D**–**F**) (*n* = 4). * *p* < 0.05, ** *p* < 0.01 vs. respective sedentary (Sed) group; ^#^ *p* < 0.05, ^##^ *p* < 0.01, ^###^ *p* < 0.001 vs. respective standard chow (SC) group.

After 17 weeks of dietary intervention, SC- and HF-fed animals displayed 16% (average weight gain = 5.6 ± 1.9 g) and 47% (average weight gain = 16.8 ± 2.6 g) weight gain, respectively (Figure 2A). Fasting glycemia was also significantly higher in HF animals compared to SC animals (Figure 3A), indicating that the HF diet caused impairment in glycemic control. This was confirmed by the HF animals displaying significantly higher blood glucose levels at all time points of the ITT compared to the SC animals (Figure 3B,C).

### 3.2. Effects of LC on BW

Animals in the HF_Sed_ group demonstrated a significant increase in total BW up to week 26 (average weight gain = 58.5 ± 4.1 g at 26-wk vs. 52.27 ± 5.8 g at 18-wk) (Figure 2A). Importantly, the HF_LC_ group had a significantly lower final BW than the HF_Sed_ group (average weight = 48.5 ± 3.6 g vs. 58.5 ± 4.1 g, respectively (Figure 2A).

### 3.3. Effects of LC on Adiposity and WAT Morphology

Inguinal adipose tissue from the HF_Sed_ group presented with enlarged WAT cells compared to the SC_Sed_ group (196.8 ± 34.7 µm vs. 84.28 ± 12.5 µm). LC was able to reverse these morphological changes as images of the inguinal WAT from the HF_LC_ group showed WAT cells with a smaller diameter compared to the HF_Sed_ group (108.3 ± 27.7µm vs. 196.8 ± 34.7 µm, respectively). Importantly, the WAT cell diameters of the HF_LC_ group were comparable to those of the SC_LC_ group (108.3 ± 27.7 µm vs. 114.6 ± 11.1 µm, respectively). There were no differences in WAT cell diameters or morphology between the SC_Sed_ and SC_LC_ groups (Figure 2B,C).

### 3.4. Effects of LC on Metabolic Markers

After 26 weeks of feeding, the HF_Sed_ group showed significantly elevated fasting glycemia (Figure 3D). Importantly, LC was able to reverse the apparent insulin-resistant state caused by the HF diet, as demonstrated by the significantly lowered glycemia under fasting conditions (Figure 3D) and during the ITT (Figure 3E,F).

### 3.5. Effects of LC on the Phosphorylation of AKT, GSK-3β, and AMPK in the Gastrocnemius Muscle

**Figure 4:**
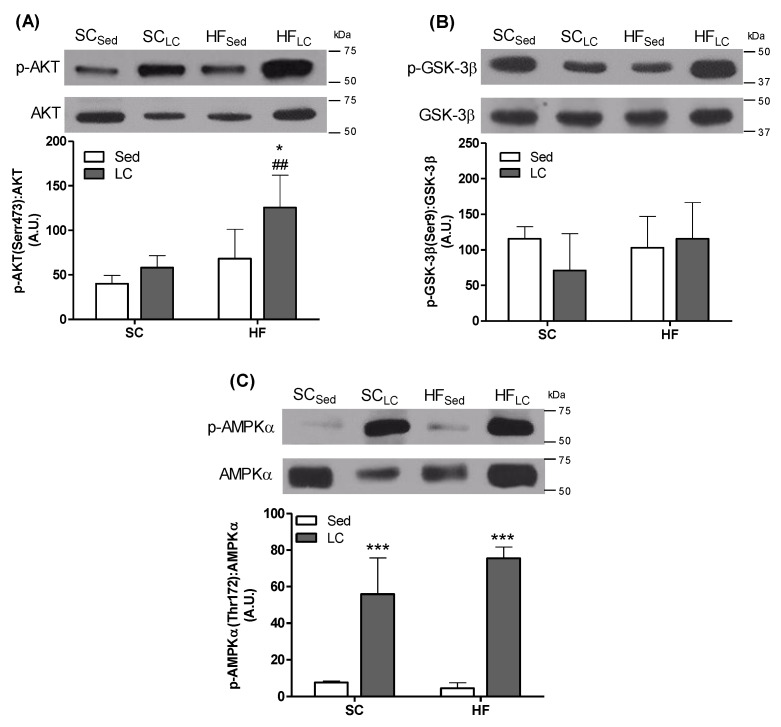
The effects of 8 weeks of LC on phosphorylation of AKT (**A**), GSK3β (**B**), and AMPKα (**C**) in the gastrocnemius muscles of mice fed either SC or HF diet. Endogenous control protein (GAPDH). Two-way ANOVA with Bonferroni posthoc test (*n* = 4). * *p* < 0.05, *** *p* < 0.001 vs. respective sedentary (Sed) group; ^##^ *p* <0.01 vs. respective SC group.

AKT phosphorylation was significantly elevated by 2-fold and 1.93-fold in the HF_LC_ group compared to the HF_Sed_ and SC_LC_ groups, respectively (Figure 4A). LC did not affect GSK-3β in the HF and RT groups (Figure 4B). However, AMPKα phosphorylation was significantly increased by 7.3-fold in the SC_LC_ group and 16.6-fold in the HF_LC_ group compared to their respective controls (Figure 4C).

### 3.6. Effects of LC on the Gene Expression of Rac1, AMPK, and CaMKK2β

**Figure 5:**
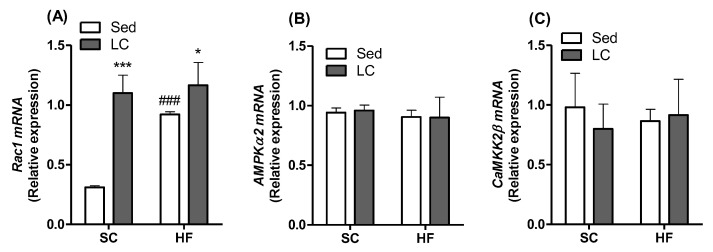
The effects of 8 weeks of LC on the gene expression of Rac1 (**A**), AMPKα2 (**B**), and CaMKK2 β (**C**) in the gastrocnemius muscles of mice fed either SC or HF diet. Data presented as relative mRNA expression. Two-way ANOVA with Bonferroni posthoc test (*n* = 4). * *p* < 0.05, *** *p* < 0.001 vs. respective sedentary (Sed) group; ^###^ *p* < 0.001 vs. respective standard chow (SC) group.

Compared to SC_Sed_ animals, all interventions elevated Rac1 gene expression (Figure 5A). Rac1 expression increased by 3.54-, 2.97-, and 3.75-fold in the SC_LC_ and HF_Sed_ groups, respectively (Figure 5A). However, Rac1 gene expression was significantly higher in the HF_LC_ group compared to the HF_Sed_ group. Neither HF nor LC had any significant effect on the gene expression of AMPKα (Figure 5B) or CaMKK2β (Figure 5C).

### 3.7. Effects of HF and LC on TNF-α and IL1β Contents and Expression of NADPH Subunits in Quadriceps Muscles

**Figure 6:**
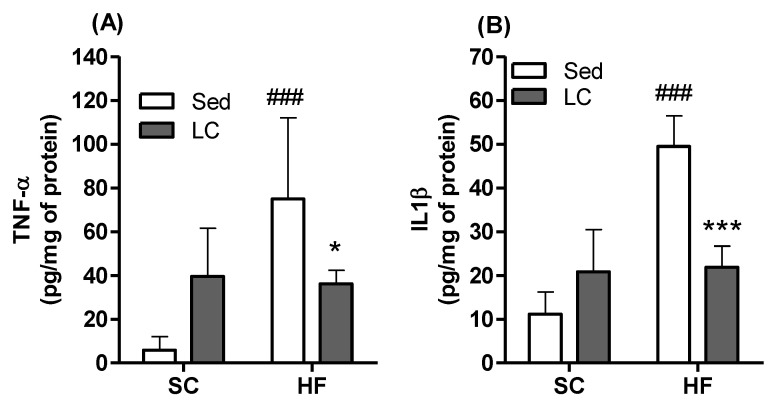
The effects of 8 weeks of LC on the levels of TNF-α (**A**) and IL1β (**B**) in the quadriceps muscles of mice fed either SC or HF diet. Two-way ANOVA with Bonferroni posthoc test *n* = 5). * *p* < 0.05, *** *p* < 0.01 vs. respective sedentary (Sed) group; ^###^ *p* < 0.001 vs. respective SC group.

**Figure 7:**
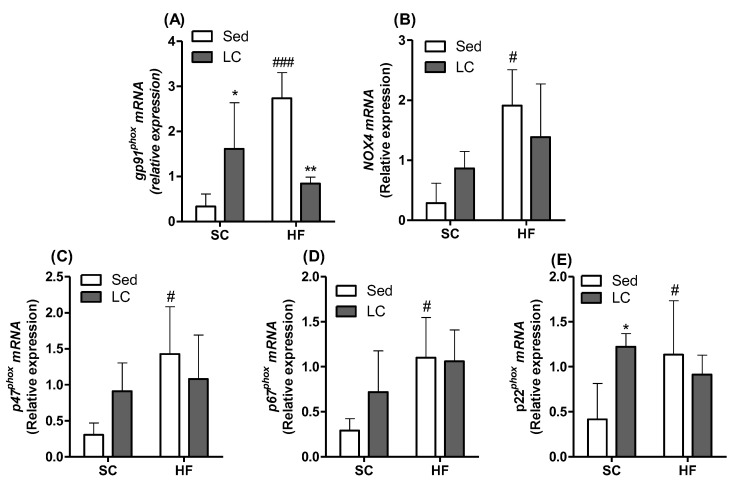
The effects of 8 weeks of LC on the gene expression of NADPH subunits in the gastrocnemius muscles of mice fed either SC or HF diet. (**A**) gp91^phox^ membrane subunit; (**B**) NOX4 membrane subunit; (**C**) p47^phox^ cytosolic subunit; (**D**) p67^phox^ cytosolic subunit; (**E**) p22^phox^ membrane/cytosolic subunit. Data presented as relative mRNA expression. Two-way ANOVA with Bonferroni posthoc test (*n* = 4). * *p* < 0.05, ** *p* < 0.01 vs. respective sedentary (Sed) group; ^#^ *p* < 0.05; ^###^ *p* < 0.001 vs. respective standard chow (SC) group.

In the HF_Sed_ group, the content of both the quadriceps muscles significantly increased by 13.8-fold and 5.31-fold, respectively, compared to SC controls. Importantly, LC was able to reverse the increase in the content of these inflammatory cytokines in the quadriceps muscles of HF mice. In fact, in the HF_LC_ group, compared to the HF_Sed_ group (TNF-α (2.25-fold) and IL1β (2.2-fold)) (Figure 6A,B), gene expression levels of components of the NADPH oxidase complex were also evaluated, and LC was shown to significantly increase the expression of the membrane gp91phox subunit (4.80-fold) (Figure 7A) and the auxiliary membrane subunit p22^phox^ (2.93-fold) (Figure 7E) in animals fed the SC. A high-fat diet (HF_Sed_) significantly increased the relative mRNA expression of all subunits evaluated when compared to the SC_Sed_ group (gp91^phox^: 8.14-fold; NOX4: 5.30-fold; p47^phox^: 4.69-fold; p67^phox^: 3.77-fold; p22^phox^: 2,72-fold) (Figure 7A–E). HF_LC_ was only able to significantly decrease the expression of the gp91^phox^ subunit compared to HF_Sed_ (3.24-fold) (Figure 7A).

## 4. Discussion

Endurance and resistance training have been used as therapeutic approaches for obesity and its associated comorbidities [46,47,48]. However, limited information is available regarding the effects of LC on adiposity, skeletal muscle inflammation, and whole-body glycemic control under conditions of diet-induced obesity. Here, we provide evidence that 8 weeks of LC prevented HF-diet-induced weight gain and reduced the adipocyte diameter in Sc Ing WAT. Additionally, HF-fed mice exposed to LC displayed fasting glycemia, similar to either Sed or LC mice fed an SC diet, and had lowered glycemia during an ITT. The ITT findings reflect whole-body insulin action and do not allow for the differentiation of the tissue-specific effects of the LC protocol. Furthermore, the response to the high insulin dose used in this protocol may reflect a scenario of elevated insulin secretion and not necessarily enhanced insulin sensitivity in peripheral tissues. However, this seems unlikely because the HF-fed sedentary obese mice, reportedly hyperinsulinemic [49,50], displayed a significantly higher glycemic response to a similar insulin dose. Thus, our results support the idea that LC training was effective in neutralizing and counteracting weight gain, attenuating inflammation, and improving insulin sensitivity in mice exposed to an HF diet. These findings are consistent with previous reports that RT increased muscle mass, reduced visceral fat, attenuated inflammation, and enhanced glucose clearance in humans and rodents [9,51,52].

LC also has the effect of reducing glucose levels by increasing the capacity of pancreatic beta-cells to secrete insulin in healthy (non-obese) animal models [53]. Although improved glycemic control is related to changes in serum insulin levels, glucose uptake is also directly related to the ability of peripheral tissues to dispose of glucose in response to insulin. It is known that in cases of insulin resistance, these mechanisms are altered [16,54]. Because LC improved insulin sensitivity in HF-fed animals, we investigated key signaling proteins involved in the regulation of insulin-stimulated glucose uptake. Indeed, HF mice exposed to LC had increased AKT phosphorylation skeletal muscle, which is consistent with an improvement in insulin sensitivity and can also be related to an increased LC-stimulated insulinemia state [53]. We expected that GSK-3β (Ser473), a downstream AKT target, would have its phosphorylation rate affected by LC; however, this was not the case. These findings are in line with previous work by Tang et al., showing that LC increased AKT phosphorylation, PI3K activity, and muscle GLUT4 expression without affecting GSK-3β phosphorylation in rats [55].

Another contributing factor to insulin-dependent glucose uptake may have been Rac1. We observed an increase in the gene expression of Rac1 in the HF_LC_ group compared to the HF_Sed_ group. Rac1 assists in the translocation of subunits of the NADPH oxidase complex from the cytosol to the membrane [56]. It also acts on the remodeling of the actin cytoskeleton, which supports the translocation of GLUT4 [16] and, consequently, glucose uptake [16,57]. Insulin-independent glucose uptake pathways were also activated following LC, as our results showed a sustained elevation in the phosphorylation of AMPKα (Thr172). In this study, none of the interventions affected *Ampk* gene expression, suggesting that this enzyme likely contributed to enhancing glucose clearance in skeletal muscle by the enhancement of its activity through phosphorylation. Additionally, because AMPK plays a crucial role in the regulation of fatty acid oxidation [58], it is also likely that its sustained elevated phosphorylation under LC conditions facilitated the utilization of fat for energy in skeletal muscles and contributed to reducing adiposity in HF-fed rats.

Excessive fat storage in obesity leads to adipose tissue dysfunction, lipotoxicity, inflammation, ectopic lipid deposition, and insulin resistance [59,60]. The low-grade chronic inflammation state in obesity is also responsible for impairment in skeletal muscle glucose uptake by activating TLR-4 and TNF-α [61]. On the other hand, the anti-inflammatory effects of physical exercise appear to be controlled by multiple mechanisms, such as the increased production of adrenaline and cortisol, among others, that have immunomodulatory effects by influencing leukocyte trafficking and functions and visceral fat loss (as described in our results) and diminishing the expression of TLRs in immune cells [62]. Furthermore, physical exercise has been demonstrated to involve a differential cytokine response, represented by increased circulating IL-6 levels, followed by a rise in IL-1ra and IL-10 levels and a suppression of TNF production [63]. We confirmed an increase in pro-inflammatory cytokines such as TNF-α and IL1β in the quadriceps muscle following HF feeding. However, these inflammatory markers were significantly reduced by LC, which likely contributed to improving insulin sensitivity and whole-body glycemic control. This is consistent with the findings of another study [64] in which the same LC exercise protocol prevented HF-diet-induced elevations in serum levels of TNF-α and IL-6 in rats.

Inflammation can also induce changes in the muscle redox profile, increasing oxidative stress [65,66,67] and potentially contributing to the development of insulin resistance [68,69]. The exact mechanism by which inflammation induces redox changes is not fully understood, but strong evidence suggests that the NADPH oxidase complex plays an important role in this process [56,70]. NADPH oxidase is an enzymatic complex that contributes to the generation of reactive oxygen species (ROS) and stimulates obesity-linked redox signaling pathways [71] and insulin resistance [67,72]. This study has shown an increased expression of the gp91phox and p22phox subunits of NADPH oxidase following LC in the SC groups. Although counterintuitive at first, an increase in NADPH oxidase subunits following exercise may result from a possible modulation of the intracellular production of superoxide and hydrogen peroxide, which are necessary for the muscular redox state following training [73]. Animals fed an HF diet also showed an increased expression of all evaluated subunits. Notably, HF_LC_ decreased the expression of gp91phox (NOX2), returning it to values similar to those of SC_Sed_ control animals. This is an important effect of LC as NOX2 has been reported to mediate muscle insulin resistance induced by an HF diet [74].

## 5. Conclusions

In summary, our data provide evidence that LC performed with concomitant consumption of an HF diet reduced adipose tissue growth, enhanced the phosphorylation of proteins involved in the insulin signaling cascade, attenuated inflammation, and promoted positive changes in the redox state of the NADPH oxidase system in skeletal muscle. These effects of LC coincided with improved whole-body insulin-mediated blood glucose clearance and glycemic control in rats. The changes attributed to LC further our understanding of the effects of LC in a state of obesity and help elucidate how this mode of exercise benefits those suffering from this chronic condition. Future directions of this problem rely on evaluating the effects of intake control (i.e., return to standard chow) concomitant with exercise, thus analyzing how the benefits of this food/energy control protocol interact with the effects demonstrated here by resistance exercise.

The limitations of the study are: biochemical and molecular analyses were performed only at the end of the study. It was not possible to verify whether exercise protected against damage from continued exposure to a high-fat diet or whether it reversed it. Another limitation is regarding glucose uptake molecular pathway analysis. It is valid to analyze the same data during an insulin-stimulated state. Future studies adopting these methodologies are necessary for a better understanding of these processes.

## Figures and Tables

**Table 1:** Primers sequence.

Gene	Gene Bank		Sequence (5′→3′)
*Ppia* [40]	NM_008907	Forward	TATCTGCACTGCCAAGACTGAATG
		Reverse	CTTCTTGCTGGTCTTGCCATTCC
*gp91^phox^*	NM_007807.5	Forward	CCAAAACCATTCGGAGGTCTTATTT
		Reverse	TGGTACTGGGCACTCCTTTATTT
*Nox 4*	NM_015760.5	Forward	CCGGGATTTGCTACTGCCTCCATC
		Reverse	ACTCCAATGCCTCCAGCCACAC
*p22^phox^*	NM_001301284.1	Forward	GCAGAGGTCCGAAAGAAGCCGA
		Reverse	ACAGCCACTGAAGGTCACACGA
*Rac1*	NM_009007.2	Forward	CCATCAAGTGTGTGGTGGTGGGA
		Reverse	AACACGTCTGTCTGCGGGTAGG
*p47^phox^*	NM_001286037.1	Forward	CGCAGGTGAACCGTATGTAA
		Reverse	CAGGAGCTTATGAATGACCTCAA
*p67^phox^*	NM_010877.5	Forward	CATGCCTGGGAACATCGTCTTT
		Reverse	GGGTGAATCCGAAGCTCAACTG
*AMPKα2* [43]	NM_178143.2	Forward	CATGGCTGAGAAGCAGAAGCAC
		Reverse	CTTAACTGCCACTTTATGGCCTG
*CaMKK2β* [44]	NM_001199676.1	Forward	CCAGGATTGTGGTGCCTGAAATC
		Reverse	ATTCTCGACCTCCTCTTCGGTCA

## Data Availability

Not applicable.
